# Spermidine-induced hypusination preserves mitochondrial and cognitive function during aging

**DOI:** 10.1080/15548627.2021.1933299

**Published:** 2021-06-09

**Authors:** Sebastian J. Hofer, YongTian Liang, Andreas Zimmermann, Sabrina Schroeder, Jörn Dengjel, Guido Kroemer, Tobias Eisenberg, Stephan J. Sigrist, Frank Madeo

**Affiliations:** a Institute of Molecular Biosciences, NAWI Graz, University of Graz, Graz, Austria; b BioTechMed-Graz, Graz, Austria; c Field of Excellence BioHealth, University of Graz, Graz, Austria; d Institute of Biology/Genetics, Freie Universität Berlin, Berlin, Germany; e Department of Biology, University of Fribourg, Fribourg, Switzerland; f Equipe Labellisée par la Ligue Contre le Cancer, Université de Paris Université Sorbonne Paris Cité, INSERM U1138, Centre de Recherche des Cordeliers, Paris, France; g Metabolomics and Cell Biology Platforms, Institut Gustave Roussy, Villejuif, France; h Pôle de Biologie, Hôpital Européen Georges Pompidou, AP-HP, Paris, France

**Keywords:** Autophagy, Drosophila, hypusination, learning, memory, mitophagy, Pink1, polyamines, spermidine

## Abstract

Spermidine is a natural polyamine, central to cellular homeostasis and growth, that promotes macroautophagy/autophagy. The polyamine pathway is highly conserved from bacteria to mammals and spermidine (prominently found in some kinds of aged cheese, wheat germs, nuts, soybeans, and fermented products thereof, among others) is an intrinsic part of the human diet. Apart from nutrition, spermidine is available to mammalian organisms from intracellular biosynthesis and microbial production in the gut. Importantly, externally supplied spermidine (via drinking water or food) prolongs lifespan, activates autophagy, improves mitochondrial function, and refills polyamine pools that decline during aging in various tissues of model organisms, including mice. In two adjacent studies, we explored how dietary spermidine supplementation enhances eEF5/EIF5A hypusination, cerebral mitochondrial function and cognition in aging *Drosophila melanogaster* and mice.

Dietary spermidine has been attributed many beneficial physiological effects in various animal models and in human epidemiological studies, including cardioprotective (in mice and humans), neuroprotective (in an SNCA/α-synuclein expressing fly model of Parkinson's disease and a fly model of age-associated cognitive decline), as well as life- and healthspan- promoting properties (flies, mice, humans). However, different molecular mechanisms of action for external spermidine supplementation have been reported, likely depending on the tissue type and experimental model. Recently, hypusination of ElF5A (eukaryotic translation initiation factor 5A) has gained attention as a direct molecular target to control spermidine-induced autophagy. In fact, this unique posttranslational modification requires spermidine as the sole and natural substrate. Hypusination occurs exclusively on a specific lysine residue in EIF5A, an essential gene in many organisms, via a 2-step enzymatic reaction (involving DHS [deoxyhypusine synthase], DOHH [deoxyhypusine hydroxylase]). Like the polyamine pathway, these genes are well-conserved across species borders. By resolving ribosomal stalling at polyproline and non-polyproline-specific motifs, hypusinated EIF5A, *inter alia*, improves translation of the key autophagic protein ATG3, which is especially important in mitochondrial homeostasis, and TFEB (transcription factor EB), a central regulator of lysosomal biogenesis and autophagy. Upon nuclear translocation, TFEB activates a transcriptional program, which has been implicated in mitochondrial quality control, including PINK1-PRKN/Parkin-mediated mitophagy, mitochondrial biogenesis via increased *PPARGC1A/PGC1-α* expression, and immunity.

In our studies [1,2], we revealed that dietary spermidine supplemented via drinking water reaches different areas of the mouse brain. Consequently, we found elevated hypusination levels in hippocampi of aged (two-year-old) mice fed with spermidine for the last 6 months, as well as in brains of spermidine-treated flies. Brains of spermidine-fed flies show higher Atg3 levels, which are unresponsive toward spermidine when the Drosophila *CG8005/DHS* was knocked down via neuron-specific RNAi. We did not detect any changes in total eEF5/EIF5A or Mitf/TFEB at the timepoints that we chose for the analyses. Thus, other yet to be determined translation targets of hypusinated eEF5/EIF5A may be involved in the spermidine effects, especially in the context of brain aging. Spermidine feeding provokes a global molecular impact on neuronal tissue, as detected by altered proteomes in aged mouse brains and increased abundance of mitochondrial proteins in the proteomic landscape of spermidine-fed fly brains. Importantly, neuronal knockdown of the Drosophila *CG8005/DHS* is sufficient to impair mitochondrial function and reduce the abundance of mitochondrial proteins, highlighting a possible role of hypusination in synthesizing certain subsets of mitochondrial proteins.

Spermidine levels decline with aging in fly heads and various other tissue types. We observed that levels of hypusinated eEF5/EIF5A also decline with age in fly brains, but not in mouse hippocampi, at least until two years of age. Concurrently, mitochondrial respiration, especially complex I-mediated oxidative phosphorylation, declines with age in both mouse and fly brains. Both the age-associated decreases in hypusination and mitochondrial function can partially be reversed by spermidine-feeding. We employed several genetic strategies to show a direct relationship between these effects: (i) loss-of-function knockouts of *Atg7, Prkn* and *Dhs*, (ii) neuronal knockdowns of *Pink1* and *Dhs*, and (iii) reduction of hypusination by substituting the target lysine with arginine in eEF5/EIF5A in a heterozygous manner. All these interventions prevent spermidine-mediated enhancement of mitochondrial respiration in flies. Whereas the chronological sequence of the involved factors is yet to be determined, our combined results suggest a cascade in which elevated dietary spermidine levels enhance eEF5/EIF5A-hypusination, which is then followed by ATG3-, and ATG7, as well as PINK1-PRKN-mediated effects on mitochondrial function. Yet, whether these factors are directly linked to translational control by hypusinated EIF5A or involve intermediate control by transcription factors or other mediators should be addressed in future studies.

We previously reported that spermidine improves memory capacity of aged flies in an autophagy-dependent fashion. It is well known that mitochondrial function and abundance are immensely important for proper synaptic function. In our recent papers we extend our understanding of these relationships by showing that spermidine-mediated improvements of cognitive function in flies likewise depend on the mitophagic mediator Pink1, as well as CG8005/DHS, implying that the abovementioned spermidine-hypusination-mitochondria axis is crucial for maintaining proper cognitive function during aging. It is noteworthy that heterozygous DHS loss of function largely occludes spermidine-mediated lifespan and locomotion improvements. Physiologically aged male (but not female) mice supplemented with spermidine perform significantly better in the Morris water maze test-based spatial learning task. This sex-specific difference needs to be studied further and may depend on a different dosing requirement (to be tested in future studies) or reflect the fact that 24-month-old female mice *per se* perform better than age-matched male mice, thus leaving little room for improvement.

Finally, data from the Bruneck study, collected between 1995 and 2000, which measured dietary spermidine intake without additional polyamine supplementation suggest an inverse correlation of intake levels and cognitive impairment in humans. This correlation withstands several adjustments for age, sex, caloric intake, social status, and education levels.

Mechanistically, several steps in the observed effects of dietary spermidine remain elusive (Figure 1). Importantly, it is still unclear how exactly the increase in mitochondrial respiration affects cognitive function or in which precise order the molecular events occur. It also remains uncertain how dietary spermidine is taken up by different cell types and how it feeds into the intracellular polyamine pathway. Last, in addition to hypusination, spermidine may act through other mechanistic routes as well, supporting autophagy-dependent or -independent mitochondrial quality control. Taken together, the sum of pre-clinical and epidemiological data highlights spermidine as an agent that might improve cognitive function in humans, calling for its clinical characterization in interventional studies.

**Figure 1.**
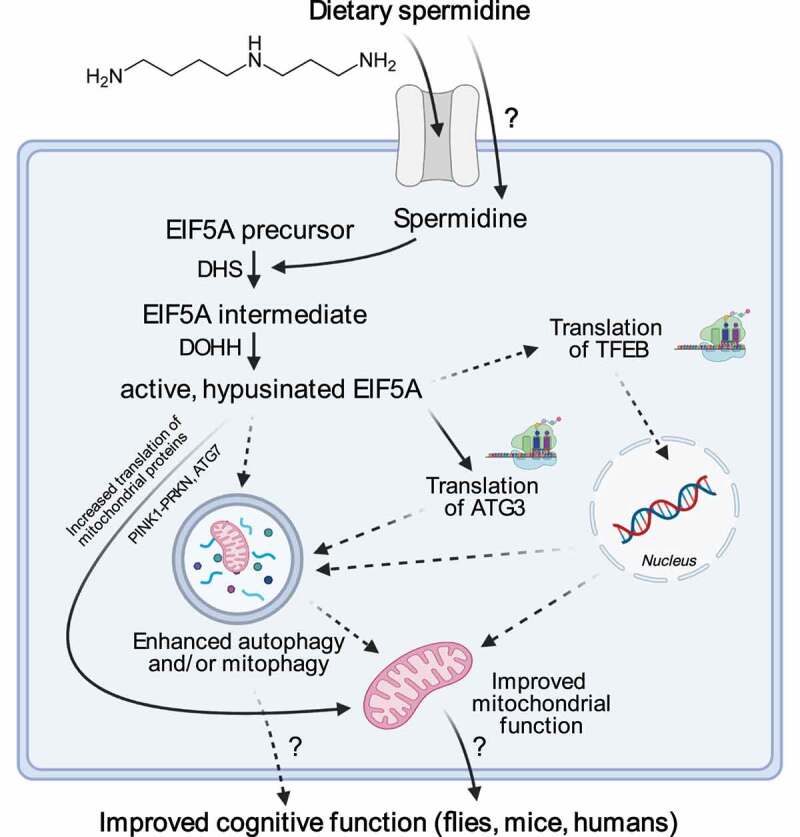
Proposed model of dietary spermidine’s effects on hypusination, mitochondria and cognitive function. Elevated dietary spermidine sets off a cascade, leading to increased levels of hypusinated eEF5/EIF5A, which orchestrates a network of effects leading to improved mitochondrial function and consequently enhanced cognitive function during aging. Dashed lines represent likely effects which were not directly measured or observed in our studies [1,2]. ATG3, autophagy related 3; DHS, deoxyhypusine synthase; DOHH, deoxyhypusine hydroxylase; EIF5A, eukaryotic translation initiation factor 5A; TFEB, transcription factor EB; PINK1, PTEN induced kinase 1
